# Fabrication and Performance of ZnO Doped Tantalum Oxide Multilayer Composite Coatings on Ti6Al4V for Orthopedic Application

**DOI:** 10.3390/nano9050685

**Published:** 2019-05-02

**Authors:** Ziyu Ding, Quanguo He, Zeliang Ding, Cuijiao Liao, Dongchu Chen, Ling Ou

**Affiliations:** 1 School of Life Sciences and Chemistry, Hunan University of Technology, Zhuzhou 412007, China; dingziyu0320@163.com; 2 School of Materials Science and Energy Engineering, Foshan University, Foshan 528000, China; chendc@fosu.edu.cn; 3 School of Mechanical Engineering, Hunan University of Technology, Zhuzhou 412007, China; xiaocuijiao@163.com; 4 Jiangsu Key Laboratory of Materials Surface Science and Technology, Changzhou University, Changzhou 213164, China; ouling24@126.com

**Keywords:** zinc oxide, tantalum oxide, Ti6Al4V, anti-inflammatory modification, antibacterial property, magnetron sputtering, corrosion resistance, adhesion strength

## Abstract

Ti6Al4V titanium alloy has been widely used as medical implant material in orthopedic surgery, and one of the obstacles preventing it from wide use is toxic metal ions release and bacterial implant infection. In this paper, in order to improve corrosion resistance and antibacterial performance of Ti6Al4V titanium alloy, ZnO doped tantalum oxide (Ta_x_O_y_) multilayer composite coating ZnO-Ta_x_O_y_/Ta_x_Oy/Ta_x_O_y_-TiO_2_/TiO_2_/Ti (ZnO-Ta_x_O_y_) was deposited by magnetron sputtering at room temperature. As a comparison, monolayer Ta_x_O_y_ coating was prepared on the surface of Ti6Al4V alloy. The morphology and phase composition of the coatings were investigated by field emission scanning electron microscopy (FE-SEM) and X-ray diffraction (XRD), the elemental chemical states of coating surfaces were investigated by X-ray photoelectron spectroscope (XPS). The adhesion strength and corrosion resistance of the coatings were examined by micro-scratch tester and electrochemical workstations, respectively. The results show that the adhesion strength of multilayer ZnO-Ta_x_O_y_ coating is 16.37 times higher than that of single-layer Ta_x_O_y_ coating. The ZnO-Ta_x_O_y_ composite coating has higher corrosion potential and lower corrosion current density than that of Ta_x_O_y_ coating, showing better corrosion inhibition. Furthermore, antibacterial test revealed that multilayer ZnO-Ta_x_O_y_ coating has a much better antibacterial performance by contrast.

## 1. Introduction

Ti6A14V titanium alloy, as a kind of ideal biomedical material, has been widely used in tooth implant, bone trauma products, artificial joints and other hard tissue substitutes or prostheses due to its excellent properties, such as biocompatibility, acceptable corrosion resistance, and comprehensive mechanical properties [1,2,3]. However, with the increase of service time, the release of Al and V ions owing to the interaction between Ti6Al4V and human body fluid may cause some toxic-relevant side effects (such as poisoning, allergies, and carcinogenesis), which eventually lead to failure of the implant [4,5,6,7]. Meanwhile, frequent bacterial infections during and after implantation can also lead to implant failure. Practically, 20% of implant failure are reported to be attributed to implant-related bacterial infections [8,9,10]. Therefore, improving the corrosion resistance and antibacterial properties of Ti6Al4V, in order that it attains an ideal anti-inflammatory modified surface and durably compatible with in vivo biological surroundings, has always been one of the research hotspots and also a factual challenge in the clinical application.

Since the corrosion behavior and bacterial infection of Ti6Al4V alloy are related to its surface properties, these features can be customized by surface coatings. In recent years, various inorganic coatings such as TiO_2_, Al_2_O_3_, SiO_2_, ZrO, Ta_2_O_5_, DLC, and HA have been used for surface modification of Ti6Al4V alloys, and their corrosion properties in vitro have also been investigated [11,12,13,14,15,16,17]. Among these coatings, tantalum oxide (Ta_x_O_y_) film has recently attracted much attention due to its advantages such as excellent corrosion resistance, good biocompatibility, and good wear resistance, as well as its ability to inhibit the growth of bacteria [18,19,20,21]. At present, various techniques for preparing Ta_x_O_y_ film have been reported, including magnetron sputtering [18,19,20,21,22], liquid phase deposition (LPD) [23,24], electrochemical deposition [25], pulsed laser deposition (PLD) [26], atomic layer deposition (ALD) [27] and sol-gel method [28,29]. It is pointed out that the magnetron sputtering, with thus fabricated films’ characteristics of high density and uniformity, high strong adhesion, low processing temperature, and easy-controlling film structure/composition, will be the ideal techniques for Ta_x_O_y_ films preparation [30,31].

Previous studies have reported that ZnO exhibits excellent antibacterial activities in various bacteria, such as *staphylococcus aureus*, *Salmonella* and *Escherichia coli* [32,33,34,35,36]. A small amount of ZnO doping into the surface layer of implant materials can obviously improve the antibacterial properties [37,38]. Furthermore, the addition of ZnO to the implant material can enhance the proliferation and differentiation of osteoblast cells, thus promoting osteogenesis [39,40]. In particular, the inhibitory effect of ZnO on bacterial growth is independent of ultraviolet radiation (UV) light, and does not change the pH of the surrounding medium [41]. More important, Zn is an essential trace element in the human body and plays an extremely important role in physiological activities such as DNA synthesis, enzymatic activity, and biomineralization [42]. All these advantages make ZnO suitable for acting as a functional agent to solve the antibacterial problem. In addition, there are few reports on ZnO doped Ta_x_O_y_ coatings prepared by magnetron sputtering in the present work.

However, the low adhesion strength of the ceramic coatings to the metal substrate may become the first obstacle to their widespread application [43]. The Mismatch of the thermal expansion coefficients (CTE) between the ceramic coatings and the metal substrate leads to the development of excessive stresses at the interface. It is well known that intermediate layer can be used to reduce the CTE mismatch between two different materials and consequently, give a better effect to the mechanical properties [44]. Thus, the multilayer films with one or more intermediate layers have been widely applied in versatile industries to improve the performance and lifespan of the components owing to its numerous advantages, such as superior bonding strength, hardness, toughness, and corrosion resistance compared to the monolayer films [45].

In this study, a novel multilayer composite coating ZnO-Ta_x_O_y_/Ta_x_O_y_/Ta_x_O_y_-TiO_2_/TiO_2_/Ti (code ZnO-Ta_x_O_y_) was prepared on the surface of Ti6Al4V alloy by magnetron sputtering at room temperature. The microstructure of the as-deposited coating was investigated by X-ray diffraction (XRD), X-ray photoelectron spectroscopy (XPS) and field emission scanning electron microscopy (FE-SEM). The wettability, adhesion strength and corrosion resistance of the composite coating was evaluated and compared by contact angle measurement, micro-scratch test, and electrochemical corrosion, respectively. For comparative purposes, these measurements were also carried out on Ta_x_O_y_ coated Ti6Al4V and uncoated Ti6Al4V. By understanding the microstructure and properties of ZnO-Ta_x_O_y_ composite multilayer coatings, our work is expected to provide a valuable reference for biomedical applications of this novel coating.

## 2. Materials and Experimental Design

### 2.1. Preparation of Coatings

**Figure 1:**
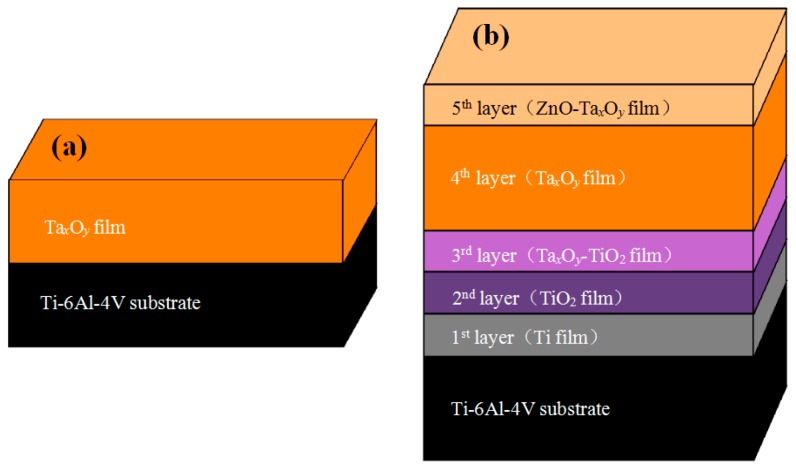
Schematic representation of ceramic composite coating structure: (**a**) Ta*_x_*O*_y_* coating, (**b**) ZnO-Ta*_x_*O*_y_* coating.

Figure 1 shows the structures of coated samples. As can be seen from the figure, ZnO-Ta*_x_*O*_y_* multilayer composite coatings consists of five layers, of which the 1st layer to 3rd layer are Ti metal layer, TiO_2_ ceramic layer and TiO_2_-Ta*_x_*O*_y_* ceramic mixed layer, respectively. Those three layers were used as the intermediate transition layers and elements penetrated at two adjacent layers interface where many elements exist together, which would be helpful to alleviate the interfacial stress and improve coating adhesion [46,47,48,49]. The 4th layer of Ta*_x_*O*_y_* is mainly provided for improving the corrosion resistance of the Ti6Al4V titanium alloy, and the 5th layer (top layer) is Ta*_x_*O*_y_* doped with ZnO which acts as an antibacterial agent. Ti6Al4V (BAOTI Group Co., Ltd., Baoji, Shaanxi, China) substrate of a thickness 0.6mm was cut to the size of 10 mm × 10 mm, and its nominal composition in wt% is: Al, 6.8; V, 4.5; Fe, 0.3; O, 0.2; C, 0.1; N, 0.05; H, 0.015, and the balance, Ti. The substrates were polished using SiC emery paper with a grit size of 300, 600, 1000, and 2000, respectively. Thereafter, the substrates were ultrasonically cleaned in acetone and alcohol, each for 15 min, then dried in a pre-vacuum dryer (ZKT-6050, Shanghai Hasuc Instrument Manufacture Co., Ltd., Shanghai, China). A high vacuum magnetron sputtering system (JCP-450, Beijing Technol Science Co., Ltd., Beijing, China) was used for plasma cleaning and depositing coating. Prior to deposition, the substrate and targets were cleaned in turn by plasma cleaning with the following process parameters: background pressure of 1.0 × 10^−3^ Pa, argon flow of 20 sccm, cleaning power of 200 W and the cleaning time of 20 min. Afterward, ZnO-Ta*_x_*O*_y_* multilayer composite coating was deposited on Ti6Al4V surfaces by sequentially depositing Ti film, TiO_2_ film, Ta*_x_*O*_y_*-TiO_2_ film, Ta*_x_*O*_y_*, and ZnO-Ta*_x_*O*_y_* film. Ta and Ti targets (Zhongnuo New Material Technology Co., Ltd. Beijing, China) had a purity of 99.99% and a size of φ 75 × 5 mm. During deposition, argon and oxygen were used as sputtering gas and reaction gas, respectively, both with the purity of 99.99%. The distance between the substrate and target was 75 mm, and the base pressure was 1 × 10^−3^ Pa. The sputtering mode, deposition parameters of coatings are shown in Table 1.

### 2.2. Characterization of Coatings

The phase composition of the coatings was analyzed by X-ray diffraction (XRD, Ultima IV, Rigaku Corporation, Tokyo, Japan) with Cu K_α_ radiation. The surface and cross-section morphologies of coated samples was investigated by a field emission scanning electron microscopy (FESEM, SU8000, Hitachi Group, Tokyo, Japan) equipped with energy dispersive X-ray spectroscopy (EDS). For taking a cross-section measurement, the coating samples were suspended in the epoxy resin, cured at 25 °C for 24 h, and then cut into 4 mm × 4 mm × 4 mm. Next, the cross-section of coating sample was ground with 600 to 2000 grit SiC paper. The elemental compositions and chemical states of coating surfaces were investigated by X-ray photoelectron spectroscope (XPS, EscaLab 250Xi, Thermo Fisher Scientific Inc., Waltham, MA, US), equipped with monochromatic Al K_α_ radiation (6 mA, 12 kV and 1486.68 eV). To remove surface contaminants, it is indispensable to sputter the surface using 2 kV Ar^+^ with raster area of 4 mm^2^ for 20 s. The scanning range was from 5° to 90°, with scan speed of 2°/min.

### 2.3. Scratch Test

Scratch tests was carried to estimate the adhesion strength of coated samples with MFT-4000 scratch tester (MFT-4000, Lanzhou Institute of Chemical Physics of the Chinese Academy of Sciences, Lanzhou, China). During scratch test, a conical diamond indenter (angle 120° and radius 200 μm) subjected to a progressive normal load from 0.1 N to 50 N moved across the surface of the coated samples with loading rate of 50 N/min. simultaneously, the variation of friction force, normal force and acoustical signal in terms of scratch distance were recorded continuously. Two critical loads, i.e., L_cl_ and L_c2_ in the scratch test were defined for the failure of the coatings. L_cl_ was the first critical load, which belongs to cohesive failure characterized by local coating puncture. With the gradual increase of normal load, the diamond indenter is passed through the coating into the substrate, cracks appear in the bottom and sides of the scratch, and the coating near the scratch appears to flake off, eventually the coatings had completely fallen off from the substrate along the scratch path. At this time, the friction force is abruptly changed, and an inflection point can be observed on the friction curve. This load force was recorded as the second critical load, L_c2_, which can be used to indicate the adhesion strength of the coatings. In order to determine the failure modes of the coating and associate them with the load at which they occurred, the scratch images were observed using an optical microscope (Seika Machinery, Inc. a subsidiary of Seika Corporation, KH-7700, Tokyo, Japan).

### 2.4. Contact Angle Measurement

The contact angles (CAs) measurements were performed to estimate the surface wettability at 20 °C and the ambient humidity (50%), using a contact angle goniometer (JC20001, Shanghai Zhongchen Digital Technology Co., Ltd., Shanghai, China) by the sessile drop method. The droplets were laid onto the sample surfaces by a standard micro-syringe, and the droplet images were captured using a camera. To obtain accurate water contact angle data, the measurements were repeated at five different locations of the samples.

### 2.5. Electrochemical Measurements

The electrochemical characteristics of the samples were detected with electrochemical workstation (SP-15/20A, Bio-Logic Science Instruments, Seyssinet-Pariset, France) with a conventional three-electrode system in which platinum sheet was used as the counter electrode (CE), a saturated Ag/AgCl electrode as the reference electrode (RE) and the samples as the working electrode (WE). The electrolyte was simulated body fluid (SBF) [50], at pH 7.4. One cm^2^ of the sample surface was exposed to the SBF solution. Potentiodynamic polarization curves were conducted in the range of −0.3~2.0 V with a scanning rate of 1 mV/s [51,52]. The corrosion potential (E_corr_) and corrosion current density (I_corr_) were calculated by the Tafel extrapolation method. All the experiments were repeated three times.

### 2.6. Antibacterial Experiment

The plate counting method is commonly used to quantitatively evaluate the antimicrobial properties of materials [53,54]. In this study, the bacterial strain of S. *aureus* (ATCC6538, Guangzhou Institute of Microbiololgy, Guangzhou, China), which is a classic strains of implant associated infections [53], was employed to analyze the antibacterial properties of coated samples in vitro by the plate counting method. Prior to the antibacterial test, all samples were placed in sealed test tubes and sterilized at 121 °C, 0.1 MPa for 30 min using a fully automatic autoclave. A series of bacterial suspension with concentrations of 10^5^–10^8^ CFU/ml using 0.9% NaCl solution were prepared for experiments. Then 60 μL of the bacterial suspension with a concentration of 10^7^ CFU/mL was put onto the sample surface, and cultured in a shaking incubator at 37 °C for 24 h. Thereafter, the sample with the suspension was placed into a sterile glass tube with 4 mL 0.9% NaCl solution and uniformly mixed with a vortex mixer. The suspension was then diluted by 100 times with 0.9% NaCl solution and mixed thoroughly again. The 200 μL of the diluted 100-fold bacterial solution was evenly spread on the counting agar plates using a screw inoculator, and then incubated in a shaking incubator at 37 °C for 24 h. Then these plates were photographed and the bacterial colonies were counted by an automatic colony imaging analysis system (Sphere Flash, Barcelona, Spain). Antimicrobial ratio (K) of the specimens was calculated using the formula [54]:

(1) $$K=\frac{A-B}{A}\times 100\%$$

where *A* and *B* was the average number of bacterial colonies (CFU/mL) for uncoated Ti6Al4V as the control group and for the coating sample, respectively. The obtained value represented an average of three test data

## 3. Results and Discussion

### 3.1. Microstructural Characterization of the Coatings

**Figure 2:**
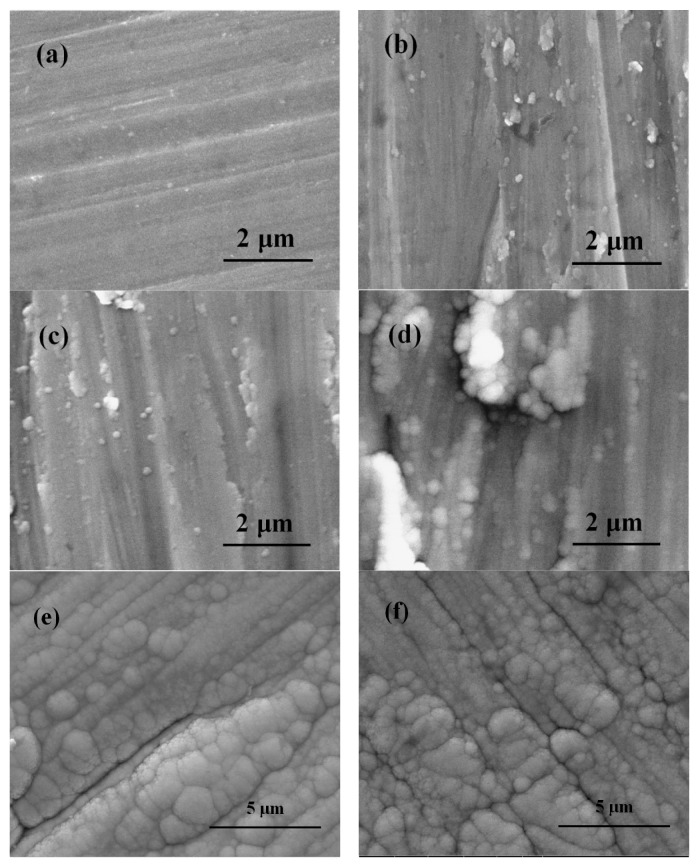
SEM images of the surface of uncoated and coated Ti6Al4V samples: (**a**) Ti6Al4V, (**b**) Ti, (**c**) TiO_2_, (**d**) Ta_x_O_y_-TiO_2_, (**e**) ZnO-Ta_x_O_y_, and (**f**) Ta_x_O_y_.

The surface morphology of the uncoated Ti6Al4V and coated Ti6Al4V are revealed in Figure 2. Figure 2a exhibits the surface characteristics of uncoated Ti6Al4V substrate after sandpaper polishing. Figure 2b–e show the surface features of the coating samples after depositing 1st layer Ti film, 2nd layer TiO_2_ film, 3rd layer Ta_x_O_y_-TiO_2_ film and 5th layer ZnO-Ta_x_O_y_ film, respectively. The surface morphology of Ta_x_O_y_/Ti6Al4V is shown in Figure 2f. In these figures, some directional grooves with different depths and widths are clearly visible, which was formed by the polishing grit. Although the groove was gradually covered by the coating with the increase of the thickness of the deposited coatings, it can still be found that there are grain boundaries or gaps with the same direction. Compared with Ta_x_O_y_ coatings, Ta_x_O_y_-TiO_2_ coating has less directional grooves, better surface smoothness, indicating a better coating quality. It is suggested that a small size abrasive should be used to polish the surface of the substrate to obtain high quality coating surface with low roughness.

**Figure 3:**
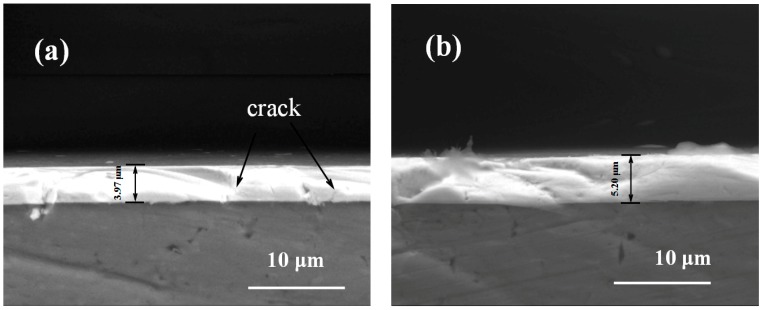
The cross-sectional SEM of coated Ti6Al4V samples of (**a**) Ta_x_O_y_ and (**b**) ZnO-Ta_x_O_y_.

Figure 3 displays the cross-section images of coated samples. The coating thicknesses of the Ta_x_O_y_ and ZnO-Ta_x_O_y_ samples were 3.97 μm (Figure 3a) and 5.2 μm (Figure 3b), respectively. As can be seen, no obvious discontinuity or crack was detected between the coating and the Ti6Al4V substrate in ZnO-Ta*_x_*O*_y_* coatings samples, indicating that the multilayer ZnO-Ta*_x_*O*_y_* coatings are well bonded to the substrate. However, some brittle cracks occur in the monolayer Ta*_x_*O*_y_* coating samples, which suggests its bonding strength to Ti6Al4V substrate is lower than that of ZnO-Ta*_x_*O*_y_* coating sample.

**Figure 4:**
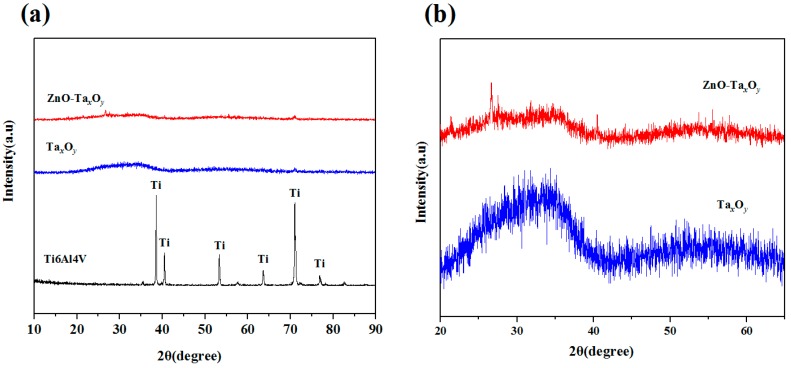
XRD patterns of samples (**a**) and coated Ti6Al4V (**b**).

The XRD spectra of uncoated and coated Ti6Al4V samples are shown in Figure 4. Compared with the XRD pattern of bare Ti-6Al-4V substrate, no characteristic peaks can be found both for Ta_x_O_y_ and ZnO-Ta_x_O_y_ films in Figure 4a, indicating an amorphous structure. The results are consistent with previous studies [20,46,47]. The reason for this amorphous nature of the deposited film may be deposition temperature [55], sputtering power [56], flow ratio of oxygen to argon [57], element doping [57], etc. The local magnification pattern shows that the diffraction pattern of Ta_x_O_y_ films is composed of diffuse-scattering curves and two humps appears in the 2θ range of 20°–65° in Figure 4b. The positions of humps coincide with the peak position of several possible tantalum oxides, such as TaO, TaO_2_ and Ta_2_O_5_ based on data in JCPDS card No.78-0724, 19-1296 and 25-0922, which signifying that the deposited Ta_x_O_y_ films may be one and more phases [47]. Besides, compared with Ta_x_O_y_ films, the diffraction curve of ZnO-Ta_x_O_y_ films becomes flat with broader hump, implying the lower crystallinity of ZnO-Ta_x_O_y_ films. The degradation of crystallinity may be caused by the incorporation of ZnO [58]. In addition, according to the data in JCPDS card NO.16-1451, the diffraction peak position of ZnO is around 31° of 2θ, and it is also in the hump area. The specific stoichiometric composition of ZnO-Ta_x_O_y_ films needs further analysis by XPS technique.

**Figure 5:**
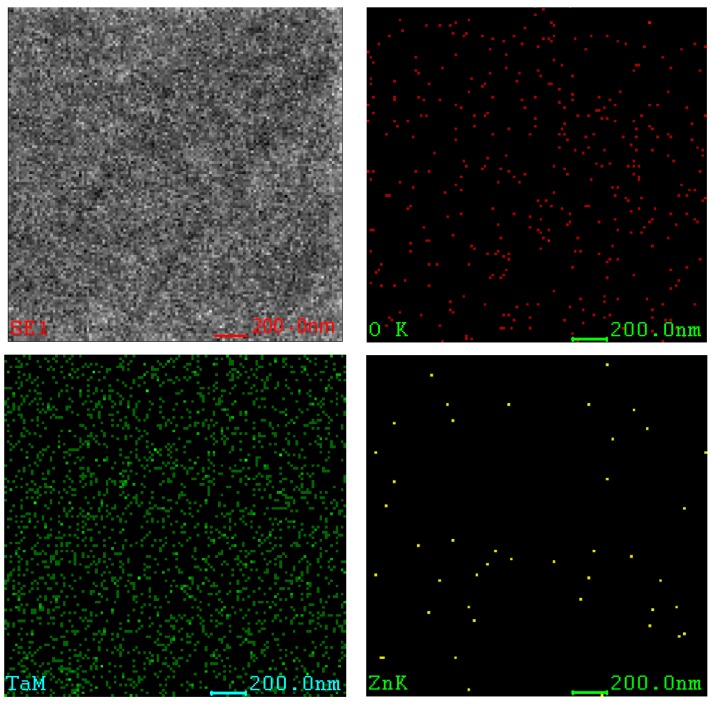
Element mapping images of ZnO-Ta_x_O_y_ coating.

Figure 5 shows the elements mapping images on the surface of ZnO-Ta_x_O_y_ coating samples. The constituent elements of the coating are Ta, Zn and O. It can be observed from the figure that each element is uniformly distributed throughout the coating. Combined with XRD analysis, the Ta element exists in its oxides, while a small amount of Zn comes from ZnO. These results show that ZnO has been incorporated into the Ta_x_O_y_ film. Ta_x_O_y_ has a significant effect on improving the corrosion resistance and biocompatibility of Ti6Al4V [1,9,10,11,12,13,14,15,16,17,18,19,20], while the presences of ZnO have a positive effect on improving the antibacterial properties of the ZnO-Ta_x_O_y_ coatings [32].

**Figure 6:**
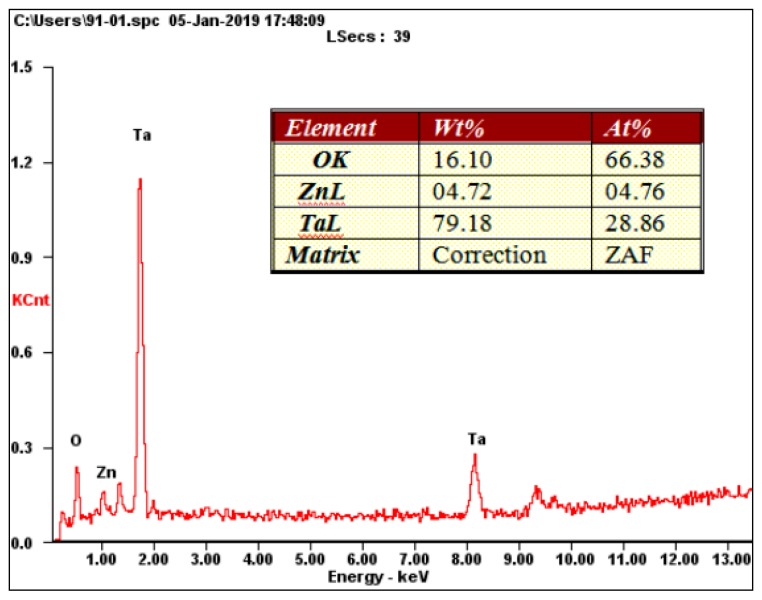
EDS element image of the ZnO-TaxOy coating.

Figure 6 shows the EDS analysis results of the ZnO-Ta_x_O_y_ coating surface. It can be seen that the content of O, Ta and Zn are approximately 16.1 wt%, 79.18 wt% and 4.72 wt% respectively. The content of each element in the coating is mainly related to the preparation parameters such as sputtering power and oxygen flow rate.

**Figure 7:**
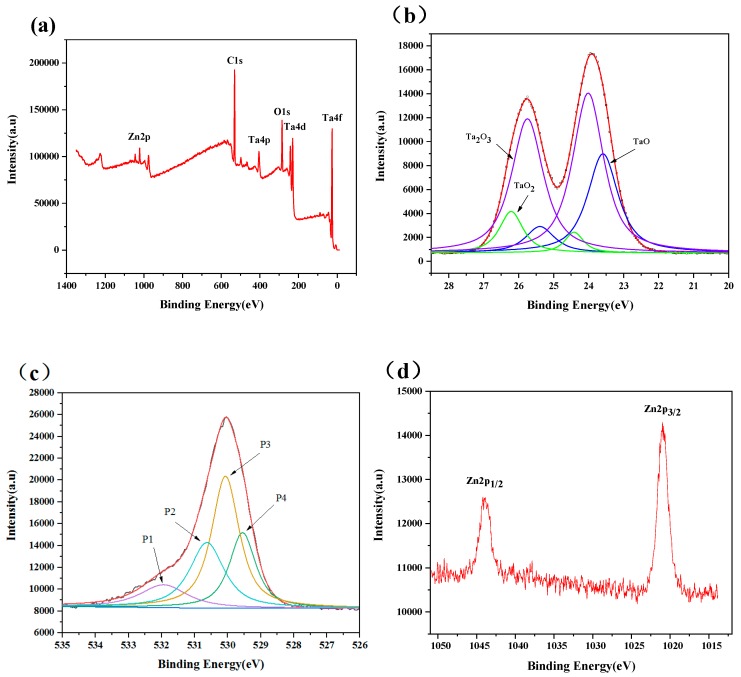
(**a**) XPS survey spectrum of ZnO-Ta_x_O_y_ coating and high-resolution spectra of (**b**) Ta 4f, (**c**) O 1s, and (**d**) Zn 2p.

Figure 7 presents the XPS survey spectrum and elemental high-resolution spectra of ZnO-Ta_x_O_y_ coated samples. It is clear that Ta, Zn and O are detected from the outermost coating of ZnO-Ta_x_O_y_ coated samples. The Ta 4f high-resolution spectrum contains three pairs of Ta 4f double peaks belonging to three chemical states of tantalum, shown in Figure 7b. The Ta 4f_7/2_ peaks associated with Ta^4+^ (TaO_2_), Ta^3+^(Ta_2_O_3_), and Ta^2+^ (TaO) are located at 24.4 eV, 24 eV, 23.6 eV respectively, and the spin-orbit splitting of Ta 4f_7/2_ to Ta 4f _5/2_ is 1.8ev, agrees with reported values [59,60,61]. The value state of Ta is related to the concentration of oxygen and the temperature during or after oxidation. The results indicate that three kinds of tantalum suboxides exist in ZnO-Ta_x_O_y_ coating surface and Ta_2_O_5_ does not appear. The binding energies of Zn 2p peak at 1021.8 eV and 1044.8 eV corresponding to Zn 2p3/2 and Zn 2p1/3 indicated that Zn element is present in the form of ZnO in the coatings [62]. As shown in Figure 7c, the O1s spectra of the coating consist of four Gaussian component peaks. The subpeak (P1) at 531.95 eV, the subpeak (P2) at 530.65 eV and subpeak (P4) at 529.55 is attributed to TaO_2_, Ta_2_O_3_, and TaO, respectively. The subpeak (P3) at 530.05 eV is assigned to ZnO. The above results show that Zn and Ta on the surface of ZnO-TaxOy coatings exist in the chemical state of ZnO and three tantalum suboxides (TaO_2_, Ta_2_O_3_ and TaO), respectively.

### 3.2. Adhesion Strength

**Figure 8:**
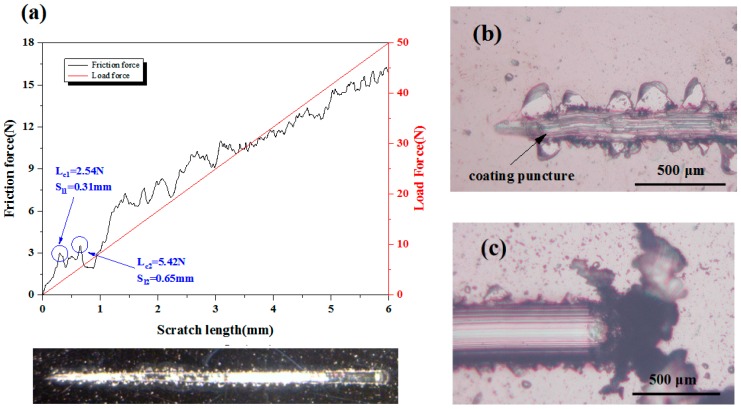
(**a**) Friction force and load force as a function of scratch length for Ta_x_O_y_ coatings, (**b**) Scratch starting position, and (**c**) scratch end.

**Figure 9:**
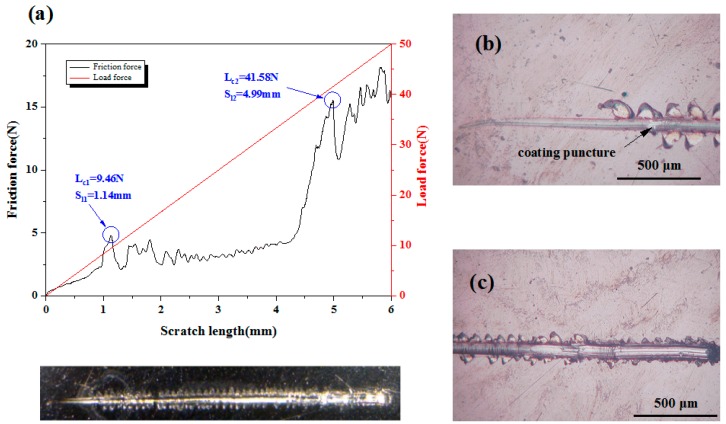
(**a**) Friction force and load force as a function of scratch length for ZnO-TaxOy coatings, (**b**) scratch starting position, and (**c**) scratch end position.

The adhesion strength between the deposited coating and substrate was investigated by scratch test. The critical loads of the scratch test were assessed by optical microscopy observations, with the help of scratch curves. As shown in Figure 8 and Figure 9, with the increase of the scratch length, the loading force increases proportionally, while the friction force shows the characteristic of oscillatory rise. The fluctuation of the friction force against scratch length may be the occurrence of film cracking and shifting due to weak interface bonding. For Ta_x_O_y_ coating samples, when the scratch length is 0.65 mm, continuous perforation and peeling of the Ta_x_O_y_ coating occurs (Figure 8b), and the critical load L_c2_ is 5.42 N (Figure 8a), which means that the binding strength of Ta_x_O_y_ is 5.42 N. Some of Ta_x_O_y_ films were accumulated at the end of the scratch, exposing the Ti6Al4V substrate along the scratch track, as shown in Figure 8c.

Figure 9 shows the scratch curve of the ZnO-Ta_x_O_y_ coating and the magnified image of scratch track. The scratch direction is from left to right. As local coating perforation was observed at the scratch distance of 1.14 mm in Figure 9b, the first critical load is 9.46 N (Figure 9a). When the scratch length is 4.99 mm, the continuous perforation appeared, with a normal load of 41.58 N, indicating that the binding strength of ZnO-Ta_x_O_y_ coating was 41.58 N. After several intermediate layers introduced into the ZnO-Ta_x_O_y_ multilayer coating, the composition changed gradually from the bulk Ti6Al4V substrate to the ZnO-Ta_x_O_y_ coating, preventing from evident interphase/interface (possible interface reaction or diffusion mechanism) [63,64] and cracks in coating (As shown in Figure 3). As a result of it, the multilayer coating demonstrated a fine binding with the substrate. What’s more, it should be noted that Ta_x_O_y_ coating and the Ti6Al4V substrate differ remarkably in thermal expansion coefficient (CTE); an environmental temperature fluctuation may cause the peel-off or dysfunction/defect of the whole coatings. The intermediate layer plays a buffering effect to reduce the CTE mismatch between the Ti6Al4V (α_Ti6Al4V_ = 8.9 × 10^−6^ K^−1^) [65] substrate and Ta_x_O_y_ coating (α_Ta-O_ = 2.9 × 10^−6^ K^−1^) [66]. Under some temperature variations, it maintains the binding ability and post heating treatment could facilitate the interfacial reactions and diffusions, in turn enhancing the adhesion strength between the Ti6Al4V substrate and the multilayer coating.

In addition, Figure 8 and Figure 9 obviously reveal that under the same experimental condition, the scratch width of ZnO-Ta_x_O_y_ sample is lower than that of TaO sample. As the coating thickness increases, the scratch resistance of coating surface enhances, and the scratch width and depth are decreased [67]. The coating thickness of ZnO-Ta_x_O_y_ sample is greater than that of sample (Figure 3), so it has higher surface resistance to scratch, and shows smaller scratch width compared with Ta_x_O_y_ sample. Further observation can be found that the first spallation occurred on the edges of scratch track near the L_C1_ position (Figure 8b and Figure 9b), and then more spallation dominated on both side of the scratch track, which revealed that the coating’s adhesive strength was higher than its cohesive strength, since spallation normally appear in brittle coatings and is induced by cracks on the coating surface or within the coating [68]. For TaO sample, the spallation appeared at a lower critical load, compared with ZnO-Ta_x_O_y_ sample, indicating that the adhesive strength of the Ta_x_O_y_ coating was lower than that of ZnO-Ta_x_O_y_ coating.

### 3.3. Corrosion Behavior

**Figure 10:**
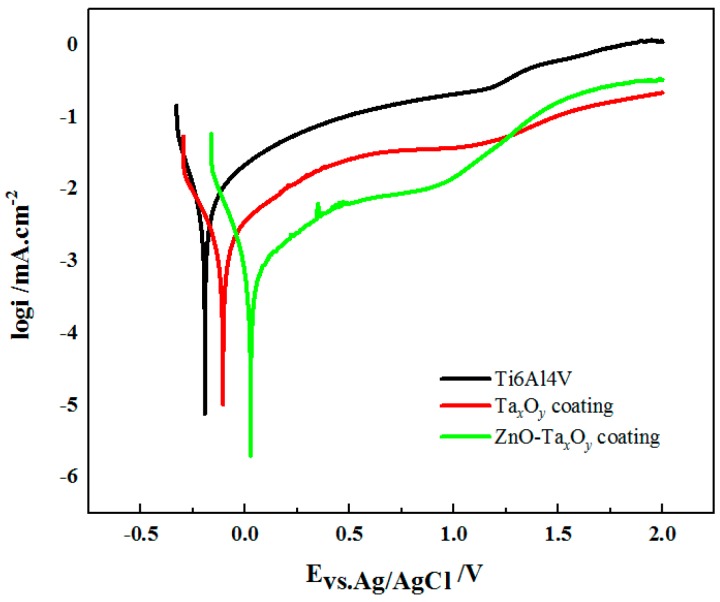
Potentiodynamic polarization curves of the uncoated and coated samples in SBF.

The representative potentiodynamic polarization curves of the un-coated and coated Ti6Al4V samples in the SBF are shown in Figure 10. The corrosion potential (E_corr_) and corrosion current density (I_corr_) derived by Tafel extrapolation method and were shown in Table 2. The corrosion potential describes the substrates’ tendency to corrode and the corrosion current density indicates the corrosion rate [18]. The corrosion potential of uncoated Ti6Al4V substrate was approximately −0.19 V, while the corrosion potential of Ta*_x_*O*_y_* and ZnO-Ta*_x_*O*_y_* coated Ti6Al4V shift towards the positive potentials, the corrosion potential of Ta*_x_*O*_y_* and ZnO-Ta*_x_*O*_y_* coated samples was −0.11 V and 0.02 V, respectively. The corrosion current densities measured from Ti6Al4V, Ta*_x_*O*_y_* and ZnO-Ta*_x_*O*_y_* coated Ti6Al4V was about 7.07 μA/cm^2^, 3.85 μA/cm^2^, and 1.12 μA/cm^2^, respectively, exhibiting a descending trend in the SBF. These indicated that the Ta*_x_*O*_y_* and ZnO-Ta*_x_*O*_y_* coatings diminish appreciably the corrosion rate of Ti6Al4V substrate. The excellent corrosion resistance of the coated samples may be concerned with the superior stability of Ta*_x_*O*_y_* ceramic coatings. Besides, the ZnO-Ta*_x_*O*_y_* coatings showed better corrosion resistance than Ta*_x_*O*_y_* coating, this can be mainly explained by the fact that ZnO-Ta*_x_*O*_y_* coatings has higher surface quality and better bond with Ti6Al4V substrate than Ta*_x_*O*_y_* coatings does. The above analyses reveal that the ZnO-Ta*_x_*O*_y_* coatings might ensure favorable anti-corrosion property in implant application.

### 3.4. Wettability

**Figure 11:**
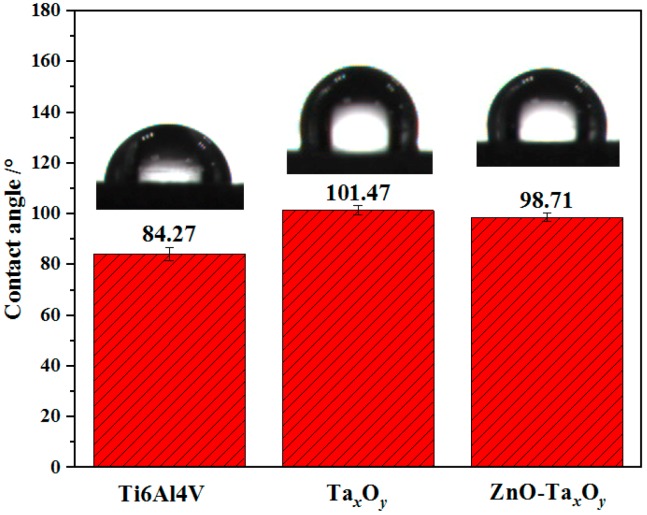
Contact angle measured for un-coated and coated Ti6Al4V samples.

Wettability refers to the ability or propensity of a liquid to spread on a solid surface. The wettability of the solid is usually evaluated by the contact angle. The smaller the contact angle, the better the wettability of the solid. Figure 11 shows the measured 4 contact angle data and the images of water drops on the surface of un-coated and coated Ti6Al4V samples. The water contact angle of Ti6Al4V alloy substrate is 84.27° ± 2.6°, indicating the hydrophilic surface. While Ta*_x_*O*_y_* and ZnO-Ta*_x_*O*_y_* coated on Ti6Al4V alloy exhibits the water contact angle greater than 90° with hydrophobic properties. The hydrophobicity of the surface helps prevent the initial adhesion of bacteria and the formation of biofilms on the surface [66,69], and is beneficial for improving the corrosion resistance of the surface.

While the hydrophobic surface can prevent cells from directly adhering on the coating surface, the amount of protein previously adsorbed on the hydrophobic surface is higher than that on the hydrophilic surface [70,71]. Protein can induce indirect cell adhesion, so the hydrophobic surface is beneficial to indirect cell adhesion. In addition, the hydrophilic surface, which was good for direct cell adhesion, actually causes side effects on protein adsorption followed by indirect cell adhesion. Therefore, the initial cell adsorption surface is neither too hydrophobic nor too hydrophilic [72]. In addition, the wettability of the surface can be controlled by doping the coating, decreasing surface roughness of the substrate, and adopting appropriate sputtering parameters [73].

### 3.5. Antibacterial Property

**Figure 12:**
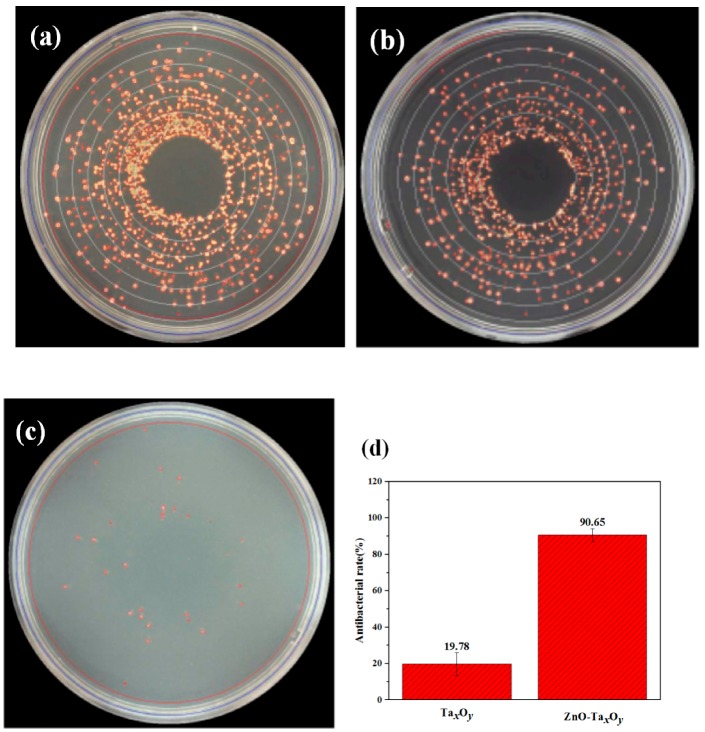
Images of *S. aureus* incubated on agar at 37 °C for 24 h on sample surface: (**a**) Ti6Al4V, (**b**) Ta_x_O_y_, and (**c**) ZnO-Ta_x_O_y_. Surface statistical of antibacterial rate (**d**).

The antibacterial properties of the ZnO-Ta*_x_*O*_y_* coating were compared with those of bare Ti6Al4V alloy and Ta*_x_*O*_y_* coating*. S. aureus* was selected as the test bacteria. Figure 12a–c shows the image of bacteriological tests of *S. aureus* on solid agar plates incubated at 37 °C for 24 h on Ti6Al4V surface, Ta*_x_*O*_y_* and ZnO-Ta*_x_*O*_y_* coatings. It is clear that there are a large number of *S. aureus* colonies incubated on Ti6Al4V surface, while several *S. aureus* colonies are observed on ZnO-Ta*_x_*O*_y_* coating. These results revealed that ZnO incorporated Ta*_x_*O*_y_* films strongly inhibited *S. aureus* growth, which had an antibacterial rate of 90.65%, while the rate for the TaxOy coating was 19.78% (Figure 12d). The antimicrobial activity of ZnO-Ta*_x_*O*_y_* coatings are due to the generation of reactive oxygen species (ROS), and release of Zn^2+^ ions [74]. The production of ROS including superoxide anion (O_2_^2−^), hydrogen peroxide (H_2_O_2_), and hydroxide (OH^−^). The O^2−^ and OH^−^ cannot penetrate into the cell membrane due to their negative charges, while H_2_O_2_ molecules are able to pass through the bacterial cell wall, subsequently leading to injuries and destruction, and finally triggering cell death [75]. The ROS produced by ZnO can kill bacteria as well as affect the growth of osteoblasts. ROS arrests osteoblasts proliferation, decrease osteoblasts growth and/or differentiation, and promotes osteoblasts death by activating various signaling [76]. The key problem of ZnO used in implant is to determine its ideal content, because of that Zn has been shown to promote osseointegration or have cytotoxic effects at low and high concentrations, respectively [77,78,79]. The released Zn^2+^ ions penetrate into the interior of the cell through the cell membrane, then interact with the genome and plasmid DNA, which interferes with the growth of the bacteria and destroys the amino acid metabolism, eventually, leading to cell death [80,81].

## 4. Conclusions

In this paper, ZnO doped tantalum oxide (Ta*_x_*O*_y_*) multilayer composite coatings (ZnO-Ta*_x_*O*_y_*/Ta*_x_*O*_y_*/Ta*_x_*O*_y_*-TiO_2_/TiO_2_/Ti, coating code ZnO-Ta*_x_*O*_y_*) were successfully fabricated on the Ti6Al4V substrate surface with magnetron sputtering technique. The results suggest that the ZnO-Ta*_x_*O*_y_* coating had great potential for improving the corrosion resistance and enhancing antibacterial property against *S. aureus* for Ti6Al4V implants. This study provides an alternative modified coating on Ti6Al4V for orthopedic application. However, the cytocompatibility and more optimization of the ZnO-Ta*_x_*O*_y_* coatings (e.g., preparation parameters, coating thickness and composition) remain to be completed in the future.

## Figures and Tables

**Table 1:** Deposition parameters.

Coating Code	Layer Number	Coating Material		Sputtering Mode	Sputtering Power (W)	Deposition Time (min)	Gas flow (sccm)	
							Ar	O_2_
ZnO-Ta*_x_*O*_y_*	1st layer	Ti		RF sputtering	200	15	16	/
	2nd layer	TiO_2_		RF reaction sputtering	200	15	16	4
	3rd layer	Ta*_x_*O*_y_*-TiO_2_	Ta*_x_*O*_y_*	DC reaction sputtering	250	15	24	6
			TiO_2_	RF reaction sputtering	200			
	4th layer	Ta*_x_*O*_y_*		DC reaction sputtering	250	120	16	4
	5th layer	ZnO-Ta*_x_*O*_y_*	ZnO	RF sputtering	150	15	16	4
			Ta*_x_*O*_y_*	DC reaction sputtering	250			
Ta*_x_*O*_y_*	/	Ta*_x_*O*_y_*		DC reaction sputtering	250	120	16	4

**Table 2:** Corrosion parameters derived from polarization curves of Figure 10.

Sample	Ti6Al4V	Ta_x_O_y_	ZnO-Ta_x_O_y_
E_corr_ (V vs. Ag/AgCl)	–0.19 ± 0.02	−0.11 ± 0.01	0.02 ± 0.01
I_corr_ (μA/cm^2^)	7.07 ± 0.012	3.85 ± 0.003	1.12 ± 0.004

## References

[1] Atar E., Kayali E.S., Cimenoglu H. (2008). Characteristics and Wear Performance of Borided Ti6Al4V alloy. Surf. Coat. Technol..

[2] Rack H.J., Qazi J.I. (2006). Titanium Alloys for Biomedical Applications. Mater. Sci. Eng. C.

[3] Gurrappa I. (2003). Characterization of Titanium Alloy Ti6Al4V for Chemical, Marine and Industrial Applications. Mater. Charact..

[4] Kamachi Mudali U., Sridhar T.M., Raj B. (2003). Corrosion of Bio Implants. Sadhana.

[5] Okazaki Y., Gotoh E. (2005). Comparison of Metal Release from Various Metallic Biomaterials in Vitro. Biomaterials.

[6] Woodman J.L., Jacobs J.J., Galante J.O., Urban R.M. (1984). Metal Ion Release from Titanium-Based Prosthetic Segmental Replacements of Long Bones in Baboons: A Long-Term Study. J. Orthop. Res..

[7] Sargeant A., Goswami T. (2007). Hip Implants—Paper VI—Ion Concentrations. Mater. Des..

[8] Raphel J., Holodniy M., Goodman S.B., Heilshorn S.C. (2016). Multifunctional coatings to simultaneously promote osseointegration and prevent infection of orthopaedic implants. Biomaterials.

[9] He Q., Liu J., Liang J., Liu X., Li W., Liu Z., Ding Z., Tuo D. (2018). Towards Improvements for Penetrating the Blood–Brain Barrier—Recent Progress from a Material and Pharmaceutical Perspective. Cells.

[10] He Q., Liu J., Liu X., Li G., Deng P., Liang J. (2018). Preparation of Cu_2_O-Reduced Graphene Nanocomposite Modified Electrodes towards Ultrasensitive Dopamine Detection. Sensors.

[11] Jiang J.Y., Xu J.L., Liu Z.H., Deng L., Sun B., Liu S.D., Wang L., Liu H.Y. (2015). Preparation, corrosion resistance and hemocompatibility of thesuperhydrophobic TiO_2_ coatings on biomedical Ti-6Al-4V alloys. Appl. Surf. Sci..

[12] Khanna R., Kokubo T., Matsushita T., Takadama H. (2016). Fabrication of dense α-alumina layer on Ti-6Al-4V alloy hybrid for bearing surfaces of artificial hip joint. Mater. Sci. Eng. C.

[13] Chellappa M., Vijayalakshmi U. (2017). Electrophoretic deposition of silica and its composite coatings on Ti-6A1-4V, and its in vitro corrosion behaviour for biomedical applications. Mater. Sci. Eng. C.

[14] Berni M., Lopomo N., Marchiori G., Gambardella A., Boi M., Bianchi M., Visani A., Pavan P., Russo A., Marcacci M. (2016). Tribological characterization of zirconia coatings deposited on Ti6Al4V components for orthopedic applications. Mater. Sci. Eng. C.

[15] Xu J., Hu W., Xie Z.H., Munroe P. (2016). Reactive-sputter-deposited β-Ta_2_O_5_ and TaON nanoceramic coatings on Ti-6Al-4V alloy against wear and corrosion damage. Surf. Coat. Technol..

[16] Hauert R., Falub C.V., Thorwarth G., Thorwarth K., Affolter C., Stiefel M., Podleska L.E., Taeger G. (2012). Retrospective lifetime estimation of failed and explanted diamond-like carbon coated hip joint balls. Acta Biomater..

[17] Kwok C.T., Wong P.K., Cheng F.T., Man H.C. (2009). Characterization and corrosion behavior of hydroxyapatite coatings on Ti6Al4V fabricated by electrophoretic deposition. Appl. Surf. Sci..

[18] Rahmati B., Sarhan A.A.D., Jeffrey Basirun W., Abas W.A.B.W. (2016). Ceramic tantalum oxide thin film coating to enhance the corrosion and wear characteristics of Ti-6Al-4V alloy. J. Alloys Compd..

[19] Hu W., Xu J., Lu X.L., Hu D.S., Tao H.L., Munroe P., Xie Z.H. (2016). Corrosion and wear behaviours of a reactive-sputter-deposited Ta_2_O_5_ nanoceramic coating. Appl. Surf. Sci..

[20] Xu J., Bao X.K., Fu T., Lyu Y.H., Munroe P., Xie Z.H. (2018). In vitro biocompatibility of a nanocrystalline β-Ta_2_O_5_ coating for orthopaedic implants. Ceram. Int..

[21] Chang Y.Y., Huang H.L., Chen H.J., Lai C.H., Wen C.Y. (2014). Antibacterial properties and cytocompatibility of tantalum oxide coatings. Surf. Coat. Technol..

[22] Bieniaś J., Surowska B., Stoch A., Matraszek H., Walczak M. (2009). The influence of SiO_2_ and SiO_2_-TiO_2_ intermediate coatings on bond strength of titanium and Ti6Al4V alloy to dental porcelain. Dental Materials.

[23] Huang A.P., Chu P.K. (2005). Crystallization improvement of Ta_2_O_5_ thin films by the addition of water vapor. J. Cryst. Growth.

[24] Huang C.J. (2005). Room-temperature formation of tantalum oxide films by liquid phase deposition. Thin Solid Film.

[25] Ispas A., Adolphi B., Bund A., Endres F. (2010). On the electrodeposition of tantalum from three different ionic liquids with the bis(trifluoromethyl sulfonyl)amide anion. Phys. Chem. Chem. Phys..

[26] Kurnia F., Hadiyawarman, Jung C.U., Jung. R. J., Liu C.L. (2011). Composition dependence of unipolar resistance switching in TaO_x_ thin films. Phys. Status Solidi RRL.

[27] Kukli K., Aarik J., Aidla A., Kohan O., Uustare T., Sammelselg V. (1995). Properties of tantalum oxide thin films grown by atomic layer deposition. Thin Solid Film.

[28] Arnould C., Korányi T.I., Delhalle J., Mekhalif Z. (2010). Fabrication of tantalum oxide/carbon nanotubes thin film composite on titanium substrate. J. Colloid Interface Sci..

[29] Wolf M.J., Roitsch S., Mayer J., Nijmeijer A., Bouwmeester H.J.M. (2013). Fabrication of ultrathin films of Ta_2_O_5_ by a sol-gel method. Thin Solid Film.

[30] Liu X.Y., Chu P.K., Ding C.X. (2004). Surface modification of titanium, titanium alloys, and related materials for biomedical applications. Mater. Sci. Eng. R Rep..

[31] Wei A.X., Ge Z.X., Zhao X.H., Liu J., Zhao Y. (2011). Electrical and optical properties of tantalum oxide thin films prepared by reactive magnetron sputtering. J. Alloys Compd..

[32] Qi K.Z., Cheng B., Yu J.G., Ho W.K. (2017). Review on the improvement of the photocatalytic and antibacterial activities of ZnO. J. Alloys Compd..

[33] Güy N., Özacar M. (2016). The influence of noble metals on photocatalytic activity of ZnO for Congo red degradation. Int. J. Hydrogen Energy.

[34] Podasca V.E., Buruiana T., Buruiana E.C. (2016). UV-cured polymeric films containing ZnO and silver nanoparticles with UV-vis light-assisted photocatalytic activity. Appl. Surf. Sci..

[35] Shim K., Abdellatif M., Choi E., Kim D. (2017). Nanostructured ZnO films on stainless steel are highly safe and effective for antimicrobial applications. Appl. Microbiol. Biotechnol..

[36] Ohtsu N., Kakuchi Y., Ohtsuki T. (2018). Antibacterial effect of zinc oxide/hydroxyapatite coatings prepared by chemical solution deposition. Appl. Surf. Sci..

[37] Sohrabnezhad S., Seifi A. (2016). The green synthesis of Ag/ZnO in montmorillonite with enhanced photocatalytic activity. Appl. Surf. Sci..

[38] Li J.H., Hong R.Y., Li M.Y., Li H.Z., Zheng Y., Ding J. (2009). Effects of ZnO nanoparticles on the mechanical and antibacterial properties of polyurethane coatings. Prog. Org. Coat..

[39] Yusa K., Yamamoto O., Fukuda M., Koyota S., Koizumi Y., Sugiyama T. (2011). In vitro prominent bone regeneration by release zinc ion from Zn-modified implant. Biochem. Biophys. Res. Commun..

[40] Kawamura H., Ito A., Miyakawa S., Layrolle P., Ojima K., Ichinose N., Tateishi T. (2000). Stimulatory effect of zinc-releasing calcium phosphate implant on bone formation in rabbit femora. J. Biomed. Mater. Res..

[41] Botequim D., Maia J., Lino M.M.F., Lopes L.M.F., Simões P.N., Ilharco L.M., Ferreira L. (2012). Nanoparticles and surfaces presenting antifungal, antibacterial and antiviral properties. Langmuir.

[42] Hu H., Zhang W., Qiao Y., Jiang X., Liu X., Ding C. (2012). Antibacterial activity and increased bone marrow stem cell functions of Zn-incorporated TiO_2_ coatings on titanium. Acta Biomater..

[43] Rahmati B., Zalnezhad E., Sarhand A.A.D., Kamiab Z., Nasiri Tabrizi B., Abasc W.A.B.W. (2015). Enhancing the adhesion strength of tantalum oxide ceramic thin film coating on biomedical Ti-6A1-4V alloy by thermal surface treatment. Ceram. Int..

[44] Zhang W.G., Liu W.M., Liu Y., Wang C.T. (2009). Tribological behaviors of single and dual sol-gel ceramic films on Ti-6Al-4V. Ceram. Int..

[45] Knotek O., Loffler F., Kramer G. (1993). Process and advantage of multicomponent and multilayer PVD coatings. Surf. Coat. Technol..

[46] Wang S.C., Liu K.Y., Huang J.L. (2011). Tantalum oxide film prepared by reactive magnetron sputtering deposition for all-solid-state electrochromic device. Thin Solid Film.

[47] Moreira H., Costa-Barbosa A., Marques S.M., Sampaio P., Carvalho S. (2017). Evaluation of cell activation promoted by tantalum and tantalum oxide coatings deposited by reactive DC magnetron sputtering. Surf. Coat. Technol..

[48] He Q., Liu J., Liu X., Li G., Deng P., Liang J. (2018). Manganese dioxide Nanorods/electrochemically reduced graphene oxide nanocomposites modified electrodes for cost-effective and ultrasensitive detection of Amaranth. Colloids Surf. B Biointerfaces.

[49] He Q., Liu J., Liu X., Li G., Chen D., Deng P., Liang J. (2019). A promising sensing platform toward dopamine using MnO_2_ nanowires/electro-reduced graphene oxide composites. Electrochim. Acta.

[50] Kokubo T., Takadama H. (2006). How useful is SBF in predicting in vivo bone bioactivity?. Biomaterials.

[51] He Q., Liu J., Liu X., Li G., Chen D., Deng P., Liang J. (2018). Fabrication of Amine-Modified Magnetite-Electrochemically Reduced Graphene Oxide Nanocomposite Modified Glassy Carbon Electrode for Sensitive Dopamine Determination. Nanomaterials.

[52] He Q., Li G., Liu X., Liu J., Deng P., Chen D. (2018). Morphologically Tunable MnO_2_ Nanoparticles Fabrication, Modelling and Their Influences on Electrochemical Sensing Performance toward Dopamine. Catalysts.

[53] Liu R., Tang Y., Zeng L., Zhao Y., Ma Z., Sun Z., Xiang L., Ren L., Yang K. (2018). In vitro and in vivo studies of anti-bacterial copper-bearing titanium alloy for dental application. Dent. Mater..

[54] Chen Y.K., Zheng X.B., Xie Y.T., Ding C.X., Ruan H.J., Fan C.Y. (2008). Anti-bacterial and cytotoxic properties of plasma sprayed silver-containing HA coatings. J. Mater. Sci. Mater. Med..

[55] Chang P.H., Liu H.Y. (1995). Structures of tantalum pentoxide thin films formed by reactive sputtering of Ta metal. Thin Solid Film.

[56] Chen H.X., Ding J.J., Shi F., Li Y.F., Guo W.G. (2012). Optical properties of Ti-doped ZnO films synthesized via magnetron sputtering. J. Alloys Compd..

[57] Jimmy Wu S.J., Houng B., Huang B.S. (2009). Effect of growth and annealing temperatures on crystallization of tantalum pentoxide thin film prepared by RF magnetron sputtering method. J. Alloys Compd..

[58] Lu J.J., Lu Y.M., Tasi S.I., Hsiung T.L., Wang H.P., Jang L.Y. (2007). Conductivity enhancement and semiconductor-metal transition in Ti-doped ZnO films. Opt. Mater..

[59] Simpson R., White R.G., Watts J.F., Baker M.A. (2017). XPS investigation of monatomic and cluster argon ion sputtering of tantalum pentoxide. Appl. Surf. Sci..

[60] Ivanov M.V., Perevalov T.V., Aliev V.S., Gritsenko V.A., Kaichev V.V. (2011). Electronic structure of Ta_2_O_5_ with oxygen vacancy: Ab initio calculations and comparison with experiment. J. Appl. Phys..

[61] Li H., Muraki Y., Karahashi K., Hamaguchi S. (2015). Suboxide/subnitride formation on Ta masks during magnetic material etching by reactive plasmas. J. Vac. Sci. Technol. A.

[62] Wang R., He X.J., Gao Y.E., Zhang X.Y., Yao X.H., Tang B. (2017). Antimicrobial property, cytocompatibility and corrosion resistance of Zn-doped ZrO_2_/TiO_2_ coatings on Ti6Al4V implants. Mater. Sci. Eng. C.

[63] Farnoush H., Aldic A., Cimenoglu H. (2015). Functionally graded HA-TiO_2_ nanostructured composite coating on Ti-6Al-4V substrate via electrophoretic deposition. Surf. Coat. Technol..

[64] Bonu V., Jeevitha M., Kumar V.P., Bysakhl S., Barshilia H.C. (2019). Ultra-thin multilayered erosion resistant Ti/TiN coatings with stress absorbing layers. Appl. Surf. Sci..

[65] Farnoush H., Mohandesi J.A., Çimenoğlu H. (2015). Micro-scratch and corrosion behavior of functionally graded HA-TiO_2_ nanostructured composite coatings fabricated by electrophoretic deposition. J. Mech. Behav. Biomed. Mater..

[66] Moldovan M., Weyant C.M., Johnson D.L., Faber K.T. (2004). Tantalum Oxide Coatings as Candidate Environmental Barriers. J. Therm. Spray Technol..

[67] Behera R.R., Das A., Pamu D., Pandey L.M., Sankara M.R. (2018). Mechano-tribological properties and in vitro bioactivity of biphasic calcium phosphate coating on Ti-6Al-4V. J. Mech. Behav. Biomed. Mater..

[68] Luo F., Gao K., Pang X., Yang H., Qiao L., Wang Y. (2008). Characterization of the mechanical properties and failure modes of hard coatings deposited by RF magnetron sputtering. Surf. Coat. Technol..

[69] Hyde F.W., Alberg M., Smith K. (1997). Comparison of fluorinated polymers against stainless steel, glass and polypropylene in microbial biofilm adherence and removal. J. Ind. Microbiol. Biotechnol..

[70] Lassen B., Holmberg K., Brink C., Carlén A., Olsson J. (1994). Binding of salivary proteins and oral bacteria to hydrophobic and hydrophilic surfaces in vivo and in vitro. Colloid Polym. Sci..

[71] Welin-Klintstrom S., Askendal A., Elwing H. (1993). Surfactant and Protein Interactions on Wettability Gradient Surfaces. J. Colloid Interface Sci..

[72] Zhou W., Zhong X., Wu X., Yuan L., Zhao Z., Wang H., Xia Y., Feng Y., He J., Chen W. (2006). The effect of surface roughness and wettability of nanostructured TiO_2_ film on TCA-8113 epithelial-like cells. Surf. Coat. Technol..

[73] Cheng Y.L., Cao L., He G., Yao G., Song X.P., Sun Z.Q. (2014). Preparation, microstructure and photoelectrical properties of Tantalum-doped zinc oxide transparent conducting films. J. Alloys Compd..

[74] Zhang L.L., Jiang Y.H., Ding Y.L., Daskalakis N., Jeuken L., Povey M., O’Neill A.J., York D.W. (2010). Mechanistic Investigation in to Antibacterial Behaviour of Suspensions of ZnO Nanoparticles Against *E. coli*. J. Nanopart. Res..

[75] Zhang L.L., Jiang Y.H., Ding Y.L., Povey M., York D. (2007). Investigation into the antibacterial behaviour of suspensions of ZnO nanoparticles (ZnO nanofluids). J. Nanopart. Res..

[76] Pandey A., Midhal S., Sharma R.K., Maurya R., Nigam V.K., Ghoshb S., Balani K. (2018). Antioxidant and antibacterial hydroxyapatite-based biocomposite for orthopedic applications. Mater. Sci. Eng. C.

[77] Ramaswamy Y., Wu C., Zhou H., Zreiqat H. (2008). Biological response of human bone cells to zinc-modified Ca-Si-based ceramics. Acta Biomater..

[78] Saha N., Keskinbora K., Suvaci E., Basu B. (2010). Sintering, microstructure, mechanical and antimicrobial properties of HAp-ZnO biocomposites. J. Biomed. Mater. Res. Part B Appl. Biomater..

[79] Zhao B.H., Zhang W., Wang D.N., Feng W., Liu Y., Lin Z., Du K.Q., Deng C.F. (2013). Effect of Zn content on cytoactivity and bacteriostasis of micro-arc oxidation coatings on pure titanium. Surf. Coat. Technol..

[80] Li M., Zhu L.Z., Lin D.H. (2011). Toxicity of ZnO Nanoparticles to Escherichia Coli: Mechanism and the Influence of Medium Components. Environ. Sci. Technol..

[81] Song W., Zhang J., Guo J., Zhang J., Ding F., Li L., Sun Z. (2010). Role of the Dissolved Zinc lon and Reactive Oxygen Species in Cytotoxicity of ZnO Nanoparticles. Toxicol. Lett..

